# First steps in automatic summarization of transcription factor properties for RegulonDB: classification of sentences about structural domains and regulated processes

**DOI:** 10.1093/database/bax070

**Published:** 2017-09-26

**Authors:** Carlos-Francisco Méndez-Cruz, Socorro Gama-Castro, Citlalli Mejía-Almonte, Marco-Polo Castillo-Villalba, Luis-José Muñiz-Rascado, Julio Collado-Vides

**Affiliations:** 1 Computational Genomics Program, Center for Genomic Sciences, National Autonomous University of Mexico, Av. Universidad, s/n, Colonia Chamilpa, Cuernavaca, Morelos 62100, Mexico

## Abstract

The RegulonDB (http://regulondb.ccg.unam.mx) team generates manually elaborated summaries about transcription factors (TFs) of *Escherichia coli* K-12. These texts involve considerable effort, since they summarize a diverse collection of structural, mechanistic and physiological properties of TFs and, due to constant new research, ideally they require frequent updating. In natural language processing, several techniques for automatic summarization have been developed. Therefore, our proposal is to extract, by using those techniques, relevant information about TFs for assisting the curation and elaboration of the manual summaries. Here, we present the results of the automatic classification of sentences about the biological processes regulated by a TF and the information about the structural domains constituting the TF. We tested two classical classifiers, Naïve Bayes and Support Vector Machines (SVMs), with the sentences of the manual summaries as training data. The best classifier was an SVM employing lexical, grammatical, and terminological features (*F*-score, 0.8689). The sentences of articles analyzed by this classifier were frequently true, but many sentences were set aside (high precision with low recall); consequently, some improvement is required. Nevertheless, automatic summaries of complete articles about five TFs, generated with this classifier, included much of the relevant information of the summaries written by curators (high ROUGE-1 recall). In fact, a manual comparison confirmed that the best summary encompassed 100% of the relevant information. Hence, our empirical results suggest that our proposal is promising for covering more properties of TFs to generate suggested sentences with relevant information to help the curation work without losing quality.

**Database URL:**

RegulonDB, http://regulondb.ccg.unam.mx

## Introduction

**Figure 1.**
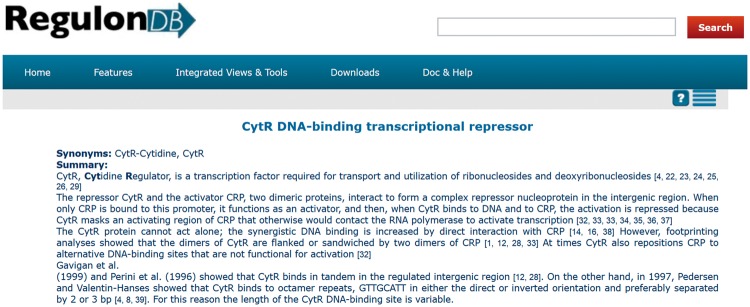
Summary of CytR from RegulonDB (http://regulondb.ccg.unam.mx/regulon?term=ECK120012407&organism=ECK12&format=jsp&type=regulon).

RegulonDB (http://regulondb.ccg.unam.mx) is a database dedicated to the transcriptional regulation of *Escherichia coli* K-12. One of the final products of curated knowledge delivered by this database is a set of summaries about several properties of transcription factors (TFs). These summaries are also found within EcoCyc (1) as part of our collaboration. Currently, we have 177 summaries. To access a summary in RegulonDB, it is necessary to search for a TF, for example, CytR. Next, we select the regulon description associated with the TF, and the summary is displayed on the top of the web page (Figure 1).

The curation team of RegulonDB developed several guidelines to create these manual summaries. These guidelines contain all the TF properties to be included, their order of appearance, some advice to describe quickly outdated information, and the requirement of incorporating references. These properties cover many relevant aspects about TFs, and they are as follows.

- The meaning of the TF acronym, indicating if it is a repressor, activator or dual transcriptional regulator.
- The function of the TF in terms of its physiological role.
- The biological processes in which the regulated genes are involved.
- The growth conditions under which the TF is expressed.
- The active and inactive conformations of the TF.
- The number, name, and size of the structural domains constituting the TF.
- Information about the TF binding site (TFBS) features (such as size and the symmetry of the consensus sequence).
- Information about the regulation mechanism that enriches the knowledge already recorded into the structured slots of the database.
- Information about evolutionary features of the TF.
- Remarks on whether the TF has other nonregulatory functions.
- Information about the organization of the transcription unit (TU) that contains the gene encoding the TF.

**Figure 2.**
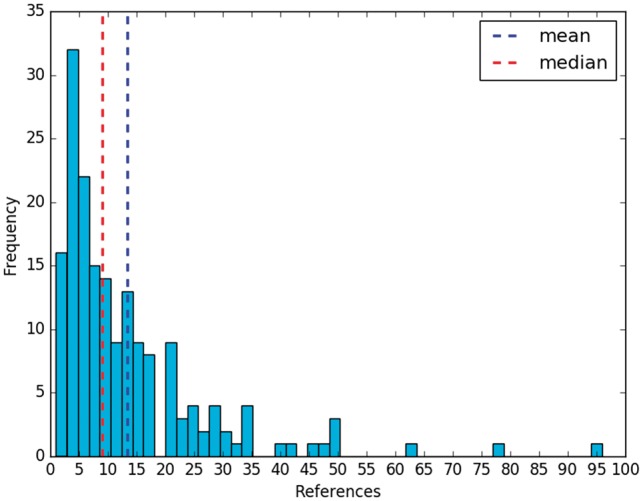
References per manual summary.

The effort to create these summaries is important. The curators of RegulonDB have already employed curated data as well as information that is not recorded in the database. In addition, they had to curate several articles, whose references were cited within each summary (Figure 1). Each manual summary includes 13 references on average, with a median of 9 (Figure 2). In relation to their size, a summary has 13 sentences and 256 words, on average.

The curation team of RegulonDB must maintain and update these manual summaries and create new summaries from new textual datasets for TFs that do not yet have one. This is because new scientific articles constantly change some aspects of the present knowledge about a biological entity, and the scientific community is eager to access new curated knowledge of new and existing organisms. The problem is that updating and writing these manual summaries is a demanding task because of the enormous growth and accelerated pace of biomedical literature and the number of features involved. Hence, clever and semiautomatic techniques to perform this task must be considered.

For some time now, natural language processing (NLP) and text mining have offered techniques to recover relevant information from document collections. One is *Automatic text summarization*, a well-established task of NLP which consists of generating a compressed version of a text collection, preserving the relevant content (2). There are, in general, two kinds of automatic summaries: extractives and abstractives. An extractive summary is made by collecting a set of relevant fragments from the text collection, whereas an abstractive summary is made by rewriting the set of extracted fragments in a shorter summary.

An automatic sumarization system for information about TFs of *E. coli* would be of great utility in accelerating the curation process. However, given the difficulty of the task, we do not currently attempt automatic generation of such summaries. Our goal is to show the initial results of partial extraction of the list of properties that could, in any case, generate suggested sentences in an assisted curation strategy (3) to accelerate the curation work without losing quality. Based on our results, we could in the future contribute to expanding our curation in scope and breadth, eventually including other organisms.

Thus, we present in this study the results of the automatic generation of summaries about only two of the features of TFs:

1. The biological processes in which the regulated genes are involved.
2. The number, name and size of the structural domains constituting the TF.

We chose these properties because they have different degrees of complexity for recovery from articles. For example, while we can find repeatedly the same controlled vocabulary to describe the structural domains of different TFs (molecular function, structural motif, domain position), the names of biological processes differ greatly among TFs. Thus, we expect that the information related to structural domains will be more easily detected than the information about regulated processes.

Thus, we proposed an initial automatic summarization strategy based on the automatic classification of sentences of articles. We trained the Support Vector Machine (SVM) and Naïve Bayes (NB) classifiers with several combinations of features extracted from sentences (words, lemmas, part-of-speech (POS) tags, term tags and frequent-word tags). For selection of the best model, we employed the sentences of the manual summaries, and for the model assessment we utilized a sample of sentences from articles manually classified by a curator. The best classifier was an SVM using unigrams and bigrams of lemmas and tags (*F*-score, 0.8689). It classified, with high precision and low recall, the sample of sentences of articles (*F*-scores, 0.45 for regulated processes, 0.47 for structural domains, 0.94 for other information).

With the selected best model, we classified the sentences of a set of complete articles on five TFs (ArgR, CytR, FhlA, GntR and MarA). Once the sentences were classified, we concatenated them to generate an initial automatic summary. We utilized the ROUGE method to evaluate the automatic summaries and determine how much relevant information they incorporated. The evaluation revealed promising results, because summaries comprised up to 76% of the relevant information of the manual summaries and up to 36% without stop words (ROUGE-1 recall). Finally, we compared manual and automatic summaries to confirm that they included much of the relevant information. For example, the worst summary according to ROUGE encompassed five out of eight relevant data elements of the manual summary, while the best one incorporated all of the relevant data. Hence, empirical results suggest that our proposal is valid to incorporate more properties of TFs and eventually generate summaries which will help the curation work and the elaboration of manual summaries for TFs which do not yet have one.

## Materials and methods

Multidocument summarization is the process used to create a single summary from a document collection (4). In this scenario, some general steps are distinguished: the retrieval of relevant documents to summarize, sentence extraction, elimination of redundancy and transformation of the summary to ensure coherence. We put the retrieval of relevant documents aside, because we exploited the set of articles used to make the manual summary by the curators instead of retrieving articles from an open collection. Nevertheless, in a broader situation, this step would need to be taken into consideration.

Regarding sentence extraction, several methods have been proposed (5, 6). The selection of one particular method depends on the purpose of the summary, among other aspects. For example, instead of producing generic summaries, we want to generate user-oriented summaries, which are made to solve a specific information need (6). For our analysis, we needed to be able to select, from a set of sentences, those sentences which contained information about the structural domain of a TF and about the biological processes in which the regulated genes are involved. This problem is a multiclass classification problem with three classes: structural domain information, regulated processes information, and other kinds of information. The task can be tackled by automatic classification techniques (supervised learning) combined with NLP. We restricted our classification problem to assign only one class per sentence (i.e. *one-of problem*), instead of assigning several classes (i.e. *any-of problem*). With this decision, although one sentence could include information about a structural domain as well as information about regulated processes, we would assign only one class.

Classification techniques have been previously employed for automatic text summarization in the biomedical domain, especially for medical question-answering systems (7–9), evidence-based medical practice (10–12) and summaries about genes (13, 14). As Sarkar *et al.* (15) pointed out, one of the main challenges in this kind of approach is that the number of positive sentences (sentences with the relevant information) is very small compared with the total number of sentences in an article.

In this study, we concentrated only on sentence classification, because the elimination of redundancy and the transformation of the summary to ensure coherence are challenging tasks by themselves. As the source of the summarization is a document collection, it is expected that some sentences will express similar information, and then a reduction of repetitive information is required. In future work, we will explore clustering techniques to avoid redundancy. Also, we plan to couple a system of sentence compression to deliver a compact summary.

## Automatic classification

Machine learning has become a useful approach to solve difficult problems in several areas (16). The idea is to have systems that can learn to make decisions about the way to solve a task. One task of machine learning is to classify objects into classes, that is, automatic classification. In this problem, a learning algorithm is fed already-classified examples of the different classes (training set) to learn decision criteria to be applied to new examples. As the input is a set of examples classified by a person, this is a case of supervised learning. Thus, the algorithm (classifier) trains a model that fits the classified examples to make predictions about novel examples. To address an automatic classification problem, we need at least the following: a classifier, classes, positive and negative examples, and features to characterize examples.

As we stated earlier, the information classes of our problem are structural domain information, regulated processes information and other kind of information. We gave one label to each class: DOM, RP and OTHER, respectively. For the DOM class, the positive examples were the sentences related to the structural domain, while the negative examples were the sentences about the RP and OTHER classes. For the RP class, the positive examples were the sentences related to regulated biological processes, whereas the negative examples were the sentences about the DOM and OTHER classes. The following subsections describe the methodology we followed, the employed classifiers, the selected features and the way we prepared the data. We have included a nomenclature guide in the Supplementary material to help the reader (Supplementary Table S11).

### Methodology

Typically, for a supervised learning problem, two tasks are performed: model selection and model assessment (17). In the former, the performance of different trained models is estimated to choose the best one by using a validation strategy, while in the latter, the level of generalization of the best model is measured on new data. To achieve these tasks, it is a common practice, in rich-data condition, to employ a random division of the available data into three data sets: training, validation and test. While the training dataset is used to fit the models, the validation dataset allows selection of the best model by estimating its performance of prediction on unseen data (data not used in training). In a different way, the test dataset is used only to assess the performance of the best model on new data, simulating a real world scenario.

Nevertheless, many real-world problems of supervised learning suffer from data scarcity, and gathering extra examples is demanding. The manual extraction of sentences about specific information is an example of this kind of problem, because curators must read several scientific articles to find a few sentences with specific information. Then, in a situation with scarce data, we can put the validation dataset aside and apply the *K-*fold cross-validation method. This method is used to estimate the performance of prediction on unseen data by using only the training dataset. The *K*-fold cross-validation method splits training data into *K* parts; it uses *K *− 1 parts to train and the remaining part for validation. It trains *K* times, always validating with a different part of the data. As the algorithm validates in every iteration with unseen data, this method gives a useful score reflecting the ability to predict (17).

**Figure 3.**
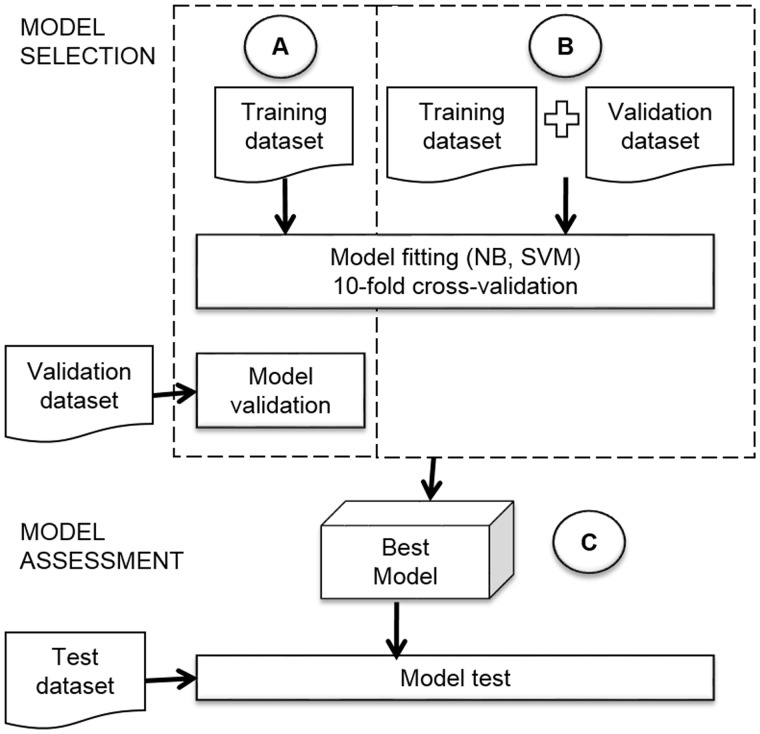
Model selection and model assessment.

In this study, we employed the two strategies explained above. In the first strategy, we fit the models of the two classifiers (NB and SVM) by 10-fold-stratified cross validation on the training dataset, and then we selected the best model with an evaluation on the validation dataset (Figure 3A). We will name this approach *strategy with validation dataset*. In the second strategy, we joined the training and validation datasets to use them for model fitting and selection with 10-fold-stratified cross validation (see Figure 3B). We name this approach *strategy with only cross-validation*. After performing the two strategies, we compared the performance of the classifiers.

The assessment of the best model (Figure 3C) had an important role in our research, because we needed to know how many sentences about structural domains and regulated processes we could obtain from articles. The assessment gave us an idea of the generalization of the model and its viability to apply in future the same strategy to new properties of TFs.

Several scores are utilized to evaluate the performance of a classifier (16–18), and three of the most frequently employed are *precision*, *recall* (sensitivity), and the *F-score* (*F*-measure). These scores are between 0 and 1, where 1 is preferred. They are based on correct and incorrect predictions of classes. Thus, there are four possible cases in a classification problem of two classes; these are represented in a confusion matrix (see Supplementary Table S12).

The precision score gives the ratio of how many examples predicted as positive are true, whereas the recall score gives the ratio of how many positive examples are predicted from all positive examples in the dataset (Equation 1). On one hand, the prediction of a classifier with high precision and low recall is frequently correct, but it sets many positive examples aside (see Classifier 1 in Supplementary Table S13). On the other hand, the decision made using a classifier with low precision and high recall is frequently incorrect, but it can classify almost all positive examples correctly, for example, assigning all the examples in the dataset (positives and negatives) to the positive class (see Classifier 2 in Supplementary Table S13).

(1) $$\begin{array}{c} \mathrm{Precision}=\frac{\text{TP}}{\text{TP}+\text{FP}}\\ \text{Recall}=\frac{\text{TP}}{\text{TP}+\text{FN}}\\ F-\text{score}=\frac{2\text{PrecisionRecall}}{\text{Precision}+\text{Recall}} \end{array}$$

Depending on the problem, it might be viable to prefer a classifier with higher precision than recall, or vice versa. Concerning our problem of classifying sentences containing specific information to be included in a summary, a classifier with high recall and low precision would result in too many false positives, which means that countless sentences with (probably) unrelated information would be curated. A classifier with high precision and low recall would get too many false negatives, that is, many lost sentences with important information. Thus, we decided to search for a classifier with a balance of precision and recall. Then, we used the *F*-score for fitting and evaluation; the *F*-score is the harmonic mean of precision and recall (Equation 1).

### Classifiers

We drew on two classical classifiers which have been demonstrated to work well in general text problems (19) and in the biomedical domain (7–9): the NB classifier (20) and SVMs (21, 22). We performed experiments with three versions of the NB classifier: Bernoulli, multinomial and Gaussian. A multinomial NB for text classification is based on the conditional probability that a document is a member of class *c* (19). Then, considering sentence s as a document, we can compute this probability as shown in Equation (2):

(2) $$P\left(c||s\right)\propto P\left(c\right)\prod_{1\leq k\leq n_{s}}P\left(f_{k}||c\right),$$

where *P*(*c*) is the prior probability of a sentence occurring in class *c*, *P*(*f_k_*|*c*) is the conditional probability of the feature *f_k_* occurring in a sentence of class *c*, and *n_s_* stands for the number of features in *s*. In general, in text classification the words of the sentences (also named terms) are the features. The best class for a sentence is the *maximum a posteriori* (MAP) class, *c*_map_, which is implemented to avoid floating point underflow by adding logarithms of probabilities. The probabilities are estimated from the already-classified examples (the training set). On one hand, *P*(*c*) is the relative frequency of class *c* in the training set (Equation 3):

(3) $$P\left(c\right)=\frac{N_{c}}{N},$$

where *N_c_* is the number of sentences in class *c* and *N* is the total number of sentences. On the other hand, *P*(*f*|*c*) is the relative frequency of the feature *f* in sentences of class *c* (Equation 4):

(4) $$P\left(f||c\right)=\frac{F_{cf}}{\sum_{f^{'}\in V}F_{cf^{'}}},$$

where *F_cf_* is the number of features *f* in sentences of class *c*, including repetitions, and the denominator is the total number of features in sentences in class *c* and *V* is the set of features. Because this probability could be 0, we add 1 to the denominator and the numerator of Equation (4); this is called Laplace smoothing.

In contrast with the multinomial classifier approach, the Bernoulli NB approach employs the presence or absence (indicated by a 1 or 0) of a feature in a sentence. This classifier estimates *P*(*f*|*c*) as the fraction of sentences of class *c* that contain the feature *f*. Regarding the Gaussian NB, the probability of the features is assumed to be Gaussian.

The second classifier is an SVM (19). This classifier type attempts to find a decision surface between two classes (internally named +1 and −1) from a vector space model representing a training dataset (Supplementary Figure S10). The distance from the decision surface to the nearest vector is called the margin (*ρ*). A maximum margin is preferred to improve certainty. For an SVM, the decision function to define the position of the class separator only depends on some points, referred as support vectors.

Given a hyperplane **w**^T^**x** = −*b*, where **w** is a normal vector, *w*^T^ is its transpose, and *b* is an intercept term related to the vertical axis, we can define an SVM linear classifier as a function which returns −1 for one class and +1 for the other class (Equation 5):

(5) $$f\left(x\right)=\text{sign}\left(w^{T}x+b\right)$$

As stated above, for an SVM classifier it is desirable to maximize the margin. Then, let ***x***_*i*_ be a vector representing a sentence and *y_i_* be a class corresponding to it; we formulated this as a minimization problem to find **w** and *b* such that:

- $1/2w^{T}w$ is minimized, and
- For all {(**x**_*i*_, *y_i_*)}, *y_i_*(**w**^T^**x**_*i*_ + *b*) ≥1

Thus, we face a quadratic optimization problem, that is, we need to optimize a quadratic function subject to linear constraints. The solution is then determined according to Equation (6):

(6) $$\begin{array}{c} w=\sum \alpha_{i}y_{i}x_{i}\\ b=y_{k}–w^{T}x_{k}\;\text{for}\;\text{any}\;x_{k}\;\text{such}\;\text{that}\;\alpha_{k}\neq 0 \end{array}$$

In this solution, each non-zero Lagrange multiplier *α_i_* indicates a support vector. Finally, the classification function of an SVM is determined according to Equation (7):

(7) $$f\left(x\right)=\text{sign}\left(\sum_{i}\alpha_{i}y_{i}x_{i}^{T}x+b\right)$$

### Features and sentence representations

Classifiers make use of features, which describe positive and negative examples, to learn classification criteria to categorize new examples into classes. Thus, the decision of which features to use is an important step. For example, if sentences of a class are represented by features which do not help to distinguish them from sentences of the other class, then the classifier is hardly going to find a classification criterion. In NLP and text mining, features are related to textual and linguistic aspects, such as lexical items, grammatical characteristics, syntactic categories and semantic representations. The selection of features involves some hypotheses of the way the objects to classify can be differentiated. Thus, we can pose the question: How can we distinguish among sentences with information about structural domains, regulated biological processes and other kinds of information?

One of the main characteristic of sentences about structural domains is the appearance of words from some controlled vocabularies, such as domain positions (*N-terminal*) and structural motifs (*helix-turn-helix*) (see Supplementary Table S14 for five randomly selected examples of sentences of each class). This is not the case for sentences about regulated processes, where the list of biological processes is quite broad. Moreover, no matter to which TF the sentences are related, many sentences share the same terms about structural domains; however, we cannot expect the same regulated biological processes for numerous TFs. This situation led us to think that categories of terms, such as *domain position*, *structural motif*, and *biological process*, could be useful as features to characterize sentences. These categories were represented by labels, commonly termed *tags*, for example, DPOS for *domain positions* and PRO for *biological processes*.

We also observed that some words (not terms) frequently appeared in sentences of one class, such as *domain* and *motif* for structural domains, and *response* for regulated processes. Similarly, these frequent words were tagged, and these tags were used as features. We used the tag FWDOM for frequent words in sentences about structural domains, and the tag FWRP for frequent words in sentences about regulated biological processes. Furthermore, some combinations of words and terms are also recurrent, like *C-terminal domain*, *N-terminal domain* and *genes involved*. Then, we posited that the combination of features could help to differentiate sentences. We employed *unigrams, bigrams* and *trigrams* of features (words and tags). A unigram is an individual feature, a bigram is a pair of consecutive features, and a trigram is formed by three contiguous features. For example, the bigrams of words obtained from the sentence ‘*SutR is a small transcription factor*’ are (‘SutR is’, ‘is a’, ‘a small’, ‘small transcription’, ‘transcription factor’).

Thus, we decided to employ five different kinds of features to represent sentences: words, lemmas, POS tags, term tags and frequent-word tags. In NLP, a lemma is a normalized (or a canonical) representation of a set of morphologically related words. For instance, the lemma of the singular and the plural form of a noun is the singular form, e.g. for *motif* and *motifs* the lemma is *motif*. In the case of verbs, the lemma is the infinitive form, that is, for *activates, activated*, and *activating*, the lemma is *activate*. Lemmas are commonly employed as features for automatic text classification because they help to match words with the same meaning but different forms. The NLP process which assigns a lemma to each word of a document is called lemmatization. Given the sentence ‘*YdeO activates genes involved in the cellular response*,’ the corresponding lemmatized sentence is ‘*YdeO****activate gene involve****in the cellular response*’.

To give the classification algorithms additional features for learning, we also considered POS tags, frequent-word tags, and term tags. A tag can be seen as a generalization of a set of specific values. For example, the tag NOUN can generalize specific nouns, such as any noun, and the tag DPOS could generalize a set of structural domain positions (*N-terminal, C-terminalcarboxy-terminal*). Thus, a classification algorithm could learn predictive patterns based on these tags in addition to learning patterns from lexical information (words or lemmas). Moreover, the frequency of a tag is higher than the frequency of the specific value, which could help classifiers. We hypothesized that these tags would be useful to find a decision criterion to classify sentences of articles.

A POS tag refers to the lexical classes (verb, noun, preposition) and grammatical categories (number, tense, mood) of words. For example, the POS of the verb *likes* is Verb, 3rd person singular present. A program named the POS tagger assigns a tag representing a part of speech to each word of a sentence. This program selects the correct tag from a set of possible tags (tag set). For example, the POS tagging for the sentence ‘*Rob is a transcriptional dual regulator*’ is *Rob_NN is_VBZ a_DT transcriptional_JJ dual_JJ regulator_NN*. The POS tags are from the Penn Treebank tag set (23): NN = noun, VBZ = verb 3rd person singular present, DT = determiner and JJ = adjective. These tags encode linguistic information that can be used by the classifier to learn patterns.

We assigned frequent-word tags only if a word belonged to one of the two sets of the 100 most frequent words in training sentences (see Supplementary Table S15, as it includes the first 15 most frequent words). To obtain these frequent words, we eliminated prepositions, conjunctions, pronouns and articles. On the one hand, if a word of a sentence appeared in the 100 most frequent words about structural domains, we assigned the tag FWDOM. On the other hand, if a word of a sentence appeared in the 100 most frequent words in sentences about regulated biological processes, we assigned the tag FWRP.

We also assigned tags to biological terms. We call these tags ‘term tags.’ A term tag was assigned if the word appeared in one of the term lists that we gathered: TFs, biological processes, molecular functions, domain positions, domain families and domain structural motifs (see the tag set in Supplementary Table S16). To obtain the list of biological processes, we generated a file in OBO format that contained the intersection of Gene Ontology’s (GO) prokaryotic subset and biological process subontology by using filters in OBO-edit (http://oboedit.org/docs/index.html). Then, we extracted only names and synonyms by using a Python script. We conserved obsolete terms, since they represent another linguistic variant to refer to a given process.

For structural domain information, we compiled some lists of terms extracted from different bioinformatics resources. We also added to these lists some terms mentioned by curators within the summaries. To obtain a list of domain families, we downloaded the datasets corresponding to TF domain assignments of *Escherichia coli* K-12 from the DNA-binding domain (DBD) Transcription Factor Prediction Database (24) (http://www.transcriptionfactor.org/index.cgi?Download) and extracted domain family names. In addition, we obtained all of the TF evolutionary families from RegulonDB (25) (http://regulondb.ccg.unam.mx/) and all of the bacterial families of proteins from Interpro (26) (https://www.ebi.ac.uk/interpro/). To obtain a list of molecular functions, we extracted GO IDs for molecular function annotations of TFs from the *E. coli* association file and got the terms corresponding to these IDs from GO’s OBO file. The domain position list was built basically from the manual summaries and was merely extended with synonyms obtained from Wikipedia. Finally, the structural motif list was extended with entries from Interpro that included the word ‘motif’ in their names.

We analyzed the lengths of terms, based on the number of words, and we found that we had terms with >5 words. This happened especially with biological processes, where we had terms with as many as 20 words. We expected that long terms were less probable to be found in articles, and then we decided to employ lists with one- to five-word terms for biological process and domain families. In the case of biological processes, terms of these lengths (one to five words) cover 80% of the list, and for domain families they cover 95%. Then, instead of incorporating the remaining terms (beyond the five words), we included their most associated collocations of three and four words. In linguistics, a collocation is a set of words which commonly appear together, such as *winding road* or *make progress*. We obtained these collocations by using the frequency of co-occurrence of words and filtering out the stop words. The stop words are words highly frequent in documents and, in general, without appreciable semantic content, such as prepositions, conjunctions, determiners and pronouns. An example of these collocations for biological processes is *acid metabolic process* obtained from terms like *downregulation of fatty acid metabolic process, upregulation of fatty acid metabolic process, cellular modified amino acid metabolic process* and *very long-chain fatty acid metabolic process*. We found that these collocations stop being terms, but they can be useful key words.

Thus, we made sentence representations by using words, lemmas, POS tags, term tags and frequent-word tags (see Table 1, rows 1–4). Also, we made some representations by combining words and lemmas with tags to give the algorithms more features to use (see Table 1, rows 5–8). We fed classifiers with these representations to determine the best one. Afterwards, we transformed the sentences of articles to this representation for applying the best classifier. However, despite this representation, we utilized the original representation of the sentences to make the automatic summaries.

**Table 1.** Features and sentence representations

Feature	Sentence representation
1 Words	ArgP, which belongs to the LysR-family, has a helix-turn-helix motif located close to the N-terminus
2 Lemmas	ArgP, which belong to the LysR-family, have a helix-turn-helix motif located close to the N-terminus
3 POS tags + Term tags	TF, r-crq VBZ p-acp dt DFAM, vdz dt DMOT NN JJ av-j p-acp dt DPOS
4 POS tags + Term tags + Frequent-word tags	TF, r-crq FWDOM p-acp dt DFAM, vdz dt DMOT FWDOM FWDOM av-j p-acp dt DPOS
5 Words + POS tags + Term tags	ArgP, which belongs to the LysR-family, has a helix-turn-helix motif located close to the N-terminus. TF, r-crq VBZ p-acp dt DFAM, vdz dt DMOT NN JJ av-j p-acp dt DPOS
6 Lemmas + POS tags + Term tags	ArgP, which belong to the LysR-family, have a helix-turn-helix motif located close to the N-terminus. TF, r-crq VBZ p-acp dt DFAM, vdz dt DMOT NN JJ av-j p-acp dt DPOS
7 Words + POS tags + Term tags + Frequent-word tags	ArgP, which belongs to the LysR-family, has a helix-turn-helix motif located close to the N-terminus. TF, r-crq FWDOM p-acp dt DFAM, vdz dt DMOT FWDOM FWDOM av-j p-acp dt DPOS
8 Lemmas + POS tags + Term tags + Frequent-word tags	ArgP, which belong to the LysR-family, have a helix-turn-helix motif located close to the N-terminus . TF, r-crq FWDOM p-acp dt DFAM, vdz dt DMOT FWDOM FWDOM av-j p-acp dt DPOS

These sentence representations were also used to obtain combinations of features in terms of *n*-grams. As we described above, a unigram is an individual feature (word, lemma, or tag), a bigram is a pair of consecutive features, and a trigram is formed by three contiguous features. For example, if we change the sentence representation based on words (Table 1, row 1) to bigrams of words, then we obtain the set (‘ArgP which’, ‘which belongs’, ‘belongs to’, ‘to the’, ‘the LysR-family’, ‘LysR-family has’, ‘has a’, ‘a helix-turn-helix’, ‘helix-turn-helix motif’, ‘motif located’, ‘located close’, ‘close to’, ‘to the’, ‘the N-terminus’). The idea behind using n-grams is to exploit contextual information, multiword expressions and collocations. To feed the classifiers, we employed unigrams, bigrams, trigrams and combinations of them: unigrams + bigrams, unigrams + bigrams + trigrams and bigrams + trigrams.

It is also common in this kind of approach based on linguistic features to wonder if highly frequent words are useful to differentiate examples. Commonly, these words are function words, such as prepositions, conjunctions, pronouns and determiners. When these kinds of words are removed, only words with semantic content remain in the sentences, for example, if we eliminate these words from the sentence ‘*It is also involved in the bacterial stringent response*,’ we obtain the sentence ‘*is also involved bacterial stringent response*.’ A positive effect of this representation is that the sentence conserves much of the sense of the original without highly frequent elements which can appear in many other sentences. However, one negative effect is that we lose useful collocations, such as *involved in*, which could be useful in some situations. The words to be removed are named *stop words*. We performed experiments with and without stop words.

Both tested classifiers received data as a Vector Space Model (19, 27). In this model, sentences are represented as vectors whose components correspond to features, for example, words. First, the set of features from all sentences is obtained (usually called vocabulary), and then the frequency of the word in the sentence is used as the value of the component (see Supplementary Figure S11). The process to convert text data into vectors is called *vectorization*.

We ran experiments with vectors of frequencies, binary values (presence/absence of features), and *tf-idf* weights. The *tf-idf* weight has been proposed in information retrieval (28) to give more importance to words that best describe a document, and it is widely used in NLP problems (18, 19). The weight is very low for terms (‘terms’ stands for words) that occur in most of the examples of a dataset, and it is higher for terms occurring in only some of them. Let *tf*(*j*) be the term frequency of the term *j* and *idf*(*j*) be the inverse document frequency of *j*, then the *tf-idf* weight of *j* is calculated as follows (Equation 8):

(8) $$\begin{array}{c} tf-idf\left(j\right)=tf\left(j\right)\times idf\left(j\right),\\ idf\left(j\right)=\text{log}\;\left(\frac{N}{df(j)}\right), \end{array}$$

where *N* stands for the number of examples in the dataset and *df*(*j*) stands for the number of examples that contain the term *j*.

It is well known that vector space models from text data are highly dimensional and sparse (18). For example, the vector space model of unigrams of lemmas and tags from our training dataset generated vectors of 3789 dimensions. For unigrams and bigrams together, the number of dimensions increased to 20 839, and when we added trigrams, the vectors reached 51 753 dimensions.

Despite the high dimensionality, we expect that a classifier can select the best features to use. However, it is feasible to find a condensed set of components from a transformation of the original vector space. This task is named *dimensionality reduction.* Benefits of this reduction are in memory, computation, complexity, reduced variance and elimination of noise and outliers (16). One of the main methods for dimensionality reduction employed in NLP is *singular value decomposition* (SVD) (19). This method is a kind of matrix decomposition that, when it is applied to text data, is usually called *Latent Semantic Analysis* (29). Let *r* be the rank of an *M* × *N* matrix **C**, then there is a SVD of **C** of the form (Equation 9):

(9) $$C=U\Sigma V^{T},$$

where *U* is an *M* × *M* matrix with orthogonal eigenvectors of *CC*^T^ as columns, *V* is the *N* × *N* matrix with orthogonal eigenvectors of *C*^T^*C* as columns, denoting *C*^T^ as the transpose of *C* and *Σ* is represented as an *r* × *r* matrix with singular values on the diagonals and all outside-diagonal entries are zeros. Thus, this singular-value decomposition is used to get a low-rank approximation *C_k_* of *C*, where *k* is a positive integer smaller than *r*. This low-rank approximation is obtained by deriving *Σ_k_* from *Σ*, replacing *r* − *k* smallest singular values with zero. Then, *C_k_* is computed as *C_k_* = UΣ*_k_V*^T^. This approximation follows the idea that the effect of small eigenvalues on matrix products is small. In practical terms, establishing a value of *k* is guided by the experimentation and is generally in the range of the low hundreds (19). Therefore, we tested dimensionality reduction to 100, 200 and 300 components.

### Datasets

At the start of our research, we were not able to obtain a dataset of sentences from articles related to structural domains of TFs and the biological processes in which the regulated genes are involved. In this situation, it is a common strategy in NLP to make a dataset with the help of a group of experts. In our case, this would have involved asking a group of curators to read a set of articles and extract sentences related to the required information. We decided not to perform this task because (i) it is demanding, and (ii) when we would need to cover all remaining properties of TFs, we would have to repeat this task for each property. Instead, we hypothesized that the classifiers could learn predictive patterns from manual summaries to classify sentences of articles. Hence, we used the sentences from existing manual summaries in RegulonDB as training examples. This decision brought a reflection. As the summaries were made by curators, they represented a limited sample of writing styles of articles. However, at the same time, they were the ideal example of sentences that we wanted to obtain, because they condensed many relevant pieces of information (they are summaries).

Therefore, 177 manual summaries were extracted from the database by the RegulonDB team and delivered to us in a single XML file. Then, we coded a Python 3.4 script to extract the text of the summaries from the XML file. Afterwards, manual summaries were tagged by a curator of RegulonDB who had participated in their elaboration, using XML-like tags to indicate text fragments related to regulated biological processes and structural domains. We employed the **<RP> **tag for regulated biological processes and the **<DOM> **tag for structural domains. The curator had full freedom to tag fragments, sentences or complete paragraphs. The result was that the curator often tagged fragments for regulated processes and the whole paragraph for structural domains (Table 2).

**Table 2.** Two examples of tagged manual summaries

CytR, Cytidine Regulator, is a TF required for **<RP>**transport and utilization of ribonucleosides and deoxyribonucleosides**</RP>** [7715459, 8596434, 8022285, 1715855, 9466254, 9767576, 14499937]
**<DOM>**ArgR has two domains: the N-terminal domain, which contains a winged helix-turn-helix DNA-binding motif [9334747] and the C-terminal domain, which contains a motif that binds L-arginine and a motif for oligomerization [8594204]. Based on cross-linking analysis of wild-type and mutant ArgR proteins, it has been shown that the C-terminus is more important in cer/Xer site-specific recombination than in DNA binding [20659168]**</DOM>**

At the end of the tagging step, we realized that the tagged fragments for regulated biological processes were very short (Table 2). We wondered if those scarce contexts would be sufficient to learn some predictive patterns. Thus, we collected all tagged fragments to set up a dataset to train the classifiers. We also created a dataset with the complete sentences where the tagged fragments appeared. If the tagged fragments coincided with complete sentences, we used those fragments as both fragments and sentences (see the last row in Table 3). Elements of the first dataset were referred to as training fragments, and those from the second set were training sentences.

**Table 3.** Examples of training fragments and training sentences

Training fragment	Training sentence
Transport and utilization of ribonucleosides and deoxyribonucleosides	CytR, Cytidine Regulator, is a TF required for transport and utilization of ribonucleosides and deoxyribonucleosides
Biosynthesis and transport of arginine, transport of histidine, and its own synthesis and activates genes for arginine catabolism	ArgR complexed with L-arginine represses the transcription of several genes involved in biosynthesis and transport of arginine, transport of histidine, and its own synthesis and activates genes for arginine catabolism
ArgR is also essential for a site-specific recombination reaction that resolves plasmid ColE1 multimers to monomers and is necessary for plasmid stability	ArgR is also essential for a site-specific recombination reaction that resolves plasmid ColE1 multimers to monomers and is necessary for plasmid stability

We attained 2237 examples for each dataset. Then, we randomly split them into training (70%) and validation (30%) datasets. We observed that the examples for regulated biological processes were fewer than examples for structural domains and that sentences about other kind of information were abundant (Table 4). This information confirms that we faced a problem of imbalanced datasets.

**Table 4.** Description of training and validation datasets

Dataset	Classes			
	DOM	RP	OTHER	Total
Training	223	190	1,153	1,566
Validation	105	70	496	671
Total	328	260	1,649	2,237

To know if the decision criterion learned by the best classifier could be generalized to sentences of articles, we created a test dataset using a set of articles of five TFs: ArgR, CytR, FhlA, GntR and MarA, whose PubMed IDs appear within manual summaries. We extracted a sample of 1019 sentences, which contained the name of the TF (Table 5). To obtain these sentences, we decided to match exactly the TF name, considering that different combinations of lower and upper cases can refer to different kinds of entities. The same curator manually classified these sentences into the DOM, RP and OTHER classes. In this dataset, the number of sentences about regulated biological processes (2%) and structural domains (8%) was much lower than the total sentences about other kinds of information (90%). In fact, except for sentences about structural domains of ArgR and MarA, there were only a few examples of each class. Moreover, in the samples, there were no sentences about regulated biological processes for FhlA.

**Table 5.** Description of the test dataset

TF	No. of articles	PMIDs	No. of sentences		
			Total	DOM	RP
ArgR	6	11305941, 1640456, 1640457, 17074904, 17850814, 8594204	216	25	9
CytR	6	10766824, 1715855, 8022285, 8596434, 8764393, 9086266	431	4	2
FhlA	5	2118503, 2280686, 8034727, 8034728, 8412675	29	4	0
GntR	7	12618441, 9045817, 9135111, 9358057, 9537375, 9658018, 9871335	194	8	3
MarA	6	10802742, 11844771, 8955629, 9097440, 9324261, 9724717	149	38	3
Total			1019	79	17

### NLP preprocessing

**Figure 4.**
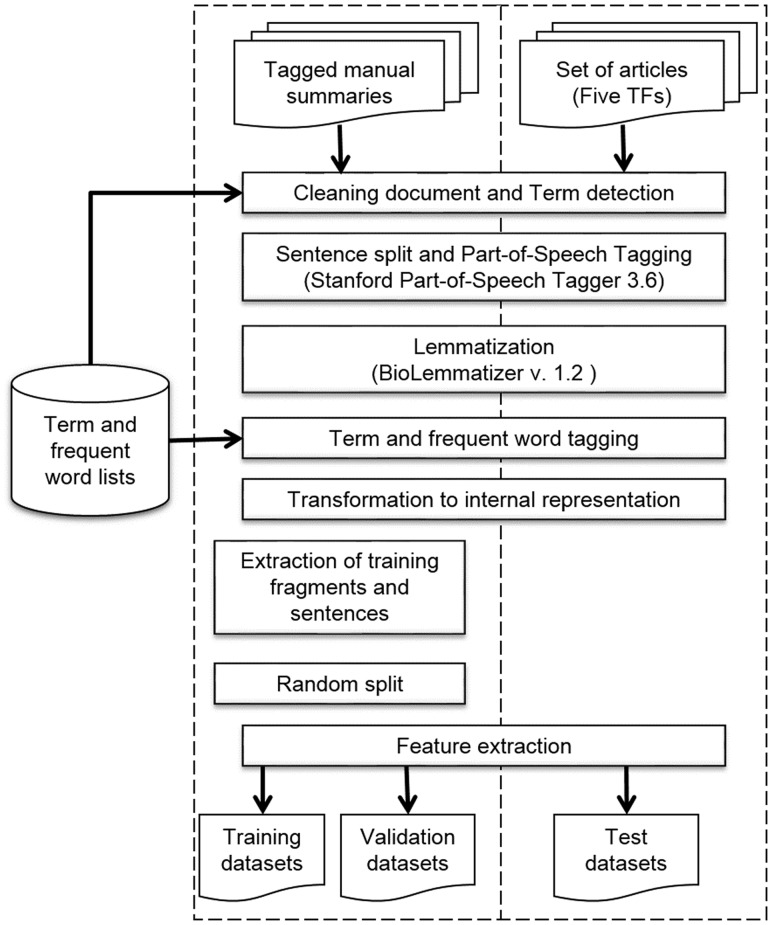
NLP preprocessing pipeline.

We built an NLP pipeline to preprocess the tagged manual summaries and the set of articles of the five TFs to obtain the sentence representations (Table 1). This pipeline included several Python 3.4 scripts and some third-party programs (https://github.com/bionlp-cgp/automatic-summarization-TFs). First, we filtered out several sections of articles, such as *Acknowledgments* and *References*, and some useless information, like author names, journal name, affiliations and the like; we named this step *Cleaning document* (Figure 4). This step did not apply to manual summaries. Next, we detected multiword terms by using the term lists. In this step, named *Term detection*, we searched for all terms within sentences. If the term matched, we joined words with hyphens. This strategy allowed us to treat multiword terms as units instead of isolated words. For example, the words *amino terminal* were detected as a term and hyphenated as *amino-terminal*.

Next, we performed *Sentence split* and *POStagging* by using the Stanford POS Tagger 3.6 program (30). This is a widely-used POS tagger which utilizes tags from the Penn Treebank tag set (23). After that, we used the BioLemmatizer 1.2 program (31) for lemmatization, which is a lemmatizer fit to the biological domain. The BioLemmatizer requires previously assigned POS tags to determine the lemmas; this was the reason we performed POS tagging before lemmatization. This lemmatizer also performs POS tagging by using the NUPOS tag set (32), which contains some more specific tags. For example, the Stanford Parser program tags the next prepositions and pronouns, such as *of_IN, about*_*IN, it_PRP* and *them*_*PRP*, whereas the BioLemmatizer tags them as *of*_*pp-f*, *about*_*IN*, *it*_*pn31* and *them*_*pno32*. We decided to utilize the tags of the BioLemmatizer for the sentence representations.

Afterwards, we accomplished *Term and frequent word tagging*. In this step, we looked for terms and frequent words within sentences; if they coincided, we replaced the POS tag with the term tag or frequent-word tag. For example, the POS-tagged sentence (a) changes to sentence (b) with term tags and to sentence (c) with frequent-word tags:

- ArgP_NN has_vdz a_dt helix-turn-helix_JJ motif_NN
- ***ArgP_TF****has_vdz a_dt****helix-turn-helix_DMOT****motif_NN*
- *ArgP_TF has_vdz a_dt helix-turn-helix_DMOT****motif_FWDOM***.

We put together words, lemmas, and tags to have an *internal representation* of each sentence; we named this step *Transformation to internal representation*. These elements were separated by a vertical bar, so an example with POS and term tags is demonstrated below in sentence (a), while an example with POS, term, and frequent-word tags is shown below in sentence (b):

- ArgP|ArgP|TF, |,|, which|which|r-crq belongs|belong|VBZ to|to|p-acp the|the|dt LysR-family|LysR-family|DFAM, |,|, has|have|vdz a|a|dt helix-turn-helix|helix-turn-helix|DMOT motif|motif|NN located|located|JJ close|close|av-j to|to|p-acp the|the|dt N-terminus|N-terminus|DPOS .|.|.
- ArgP|ArgP|TF, |,|, which|which|r-crq belongs|belong|FWDOM to|to|p-acp the|the|dt LysR-family|LysR-family|DFAM, |,|, has|have|vdz a|a|dt helix-turn-helix|helix-turn-helix|DMOT motif|motif|FWDOM located|located|FWDOM close|close|av-j to|to|p-acp the|the|dt N-terminus|N-terminus|DPOS .|.|.

Two internal representations were required, because we fit models with and without frequent-word tags to observe if these tags improved the performance of classifiers. As for the tagged manual summaries, before final step of feature extraction we obtained the training fragments and training sentences (*Extraction of training fragments and sentences*). Subsequently, we randomly split these training sets to obtain the validation datasets (*Random split*). In the case of the sentences of the set of articles on the five TFs, all sentences directly became the test dataset.

At this point, we had the datasets formed by sentences in the internal representation; then, we performed a *Feature extraction* step to build all the sentence representations mentioned above, which corresponded to different combinations of features (Table 1). Finally, we obtained eight different training and validation datasets, one for each combination of features. These datasets were subjected to classification algorithms for the learning task. Once the best combination of features was selected, we used that combination to obtain the test dataset to perform model assessment.

### Experimental setup

Following the methodology described above, we developed some experiments to elucidate whether classification with training fragments performed better than classification with training sentences. Also, we wanted to find the best classifier and the best combination of features among the eight different ones. The experimental grid with the different aspects that we tested is depicted in Table 6.

**Table 6.** Experimental grid

Aspect	Values
Classifiers	SVM, Multinomial NB, Bernoulli NB, Gaussian NB
Features	Words, Lemmas, POS tags, Term tags, Frequent-word tags
Eliminate stop words	Yes, No
*N*-grams (*n* =)	1, 2, 3, 1 + 2, 1 + 2 + 3, 2 + 3
Dimensionality reduction (SVD)	100, 200, 300 components
Vector values	Frequency, binary, TF-IDF

We coded a Python 3.4 script to perform the model selection and model assessment. We used the Python Library for Machine Learning scikit-learn version 0.18.1 (http://scikit-learn.org/stable/), which includes implementations of the SVM and NB classifiers. We accomplished the vectorization of the datasets by means of two scikit-learn objects: *CountVectorizer* and *TfidfVectorizer*. The former obtains vectors of frequencies or binary values, whereas the latter produces vectors with *tf-idf* weights. We performed dimensionality reduction of these vectors to 100, 200 and 300 components by using the *TruncatedSVD* object of scikit-learn.

We fit both classifiers by using a *RandomizedSearchCV* object. This object has a *fit* method to optimize the hyperparameters of each classifier by randomly searching over fixed values or values sampled from a given distribution. We gave the hyperparameter settings to the RandomizedSearchCV by using a Python dictionary to indicate an exponential distribution in the case of continuous values.

As for the SVM, we optimized Kernel and its parameters (see Supplementary Table S17) (http://scikit-learn.org/stable/modules/svm.html#kernel-functions), the C penalty factor (16), and the hyperparameter *class_weight*, which automatically balances the weights of each class inversely proportional to class frequencies. This hyperparameter was used to deal with the imbalanced distribution of classes. For example, of the 1153 total sentences in the training dataset, 223 sentences were of the structural domain class and only 190 were of the regulated processes class. In the test set, from 149 sentences which mentioned MarA, only 38 were about structural domains and 3 were about regulated processes. On the other hand, we optimized the hyperparameter alpha for Laplace smoothing of the Bernoulli and multinomial NB classifiers (see Supplementary Table S17).

As stated above, the optimization (model fitting) was driven by the 10-fold-stratified cross-validation of the training data with the *F*-score as the evaluation metric. Also, we established the number of iterations for the RandomizedSearchCV at 50. This is the number of parameter combinations that were iteratively sampled. With more iterations, the performance of the classifiers may improve, but the run time of the script increases significantly.

Regarding the strategy for the validation dataset, we used the models fitted by the RandomizedSearchCV to classify the validation dataset and obtain performance scores. This was done by using the *predict* method of the RandomizedSearchCV and several objects of scikit-learn to calculate precision, recall, and *F*-scores. In relation to the strategy with only cross-validation, once the RandomizedSearchCV optimized the hyperparameters of all classifiers, we obtained the best scores to be compared with the scores of the other strategy.

### Automatic summarization

**Figure 5.**
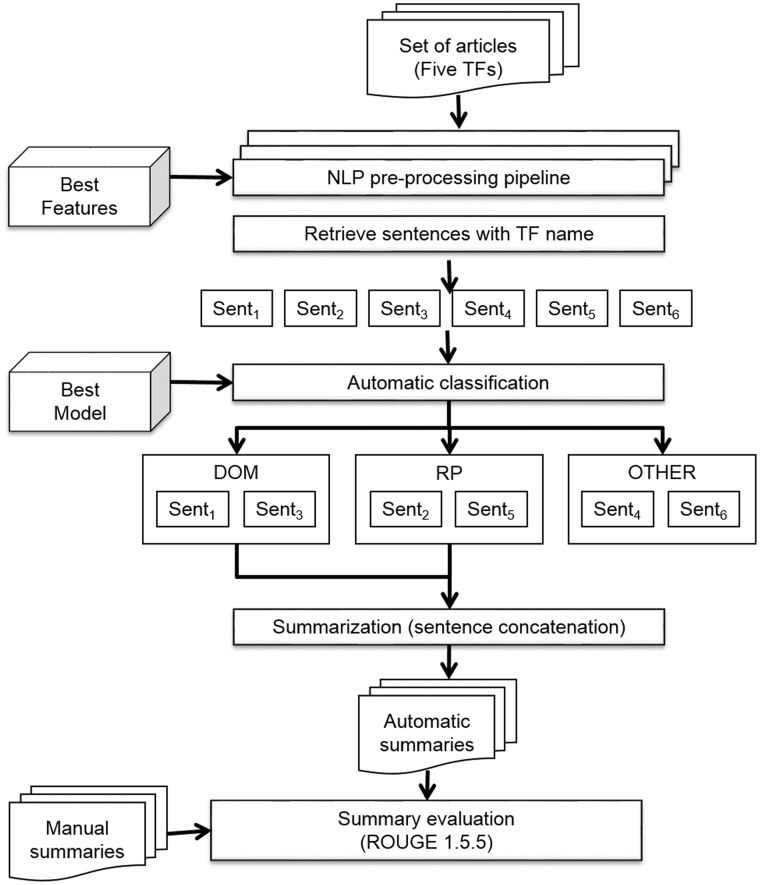
Automatic summarization pipeline.

In this study, we produced initial summaries by concatenating the sentences classified by the best classification model. We developed an automatic summarization pipeline to obtain these summaries (Figure 5). This was also coded within Python 3.4 (https://github.com/bionlp-cgp/automatic-summarization-TFs). The input of this pipeline was the set of complete articles in text format of the same five TFs (ArgR, CytR, FhlA, GntR and MarA).

First, the articles were preprocessed with the NLP preprocessing pipeline described above (see Figure 4). This pipeline returned a set of sentences represented with the best features chosen in the model selection task. Subsequently, only those sentences which included the name of the TF were retrieved. This criterion is important, on one hand, for the curation process because the curators must know the TF that the sentence describes, and on the other hand, because it ensures that sentences of the automatic summary refer to the same TF. After that, the best model was applied to automatically classify the sentences in three classes: structural domains (DOM), regulated biological processes (RP), and other kinds of information (OTHER). Only sentences of classes DOM and RP were concatenated for the final summary.

Once the automatic summary was created, we wanted to know if it resembled summaries made by the curators of RegulonDB. If this were true, we could eventually extend our methodology to the remaining features of the TFs and then generate summaries for TFs that did not have one. In the research about automatic summarization, various evaluation methods have been proposed (6, 33, 34). Evaluating an automatic summary is a difficult task because many factors are involved: the goal of the summary, the level of rewording, the level of generalization to summarize and so on. However, the widely used method is still ROUGE (we employed ROUGE 1.5.5), due to its high correlation with scores assigned by humans about the quality of automatic summaries (33).

This method, which evaluates an automatic summary compared with a manual summary, is based on the n-grams of words of the automatic summary co-occurring in the manual summary. If we divide the number of co-occurrences over the total *n*-grams of the manual summary, we obtain a score of recall, whereas if we divide the number of co-occurrences over the total *n*-grams of the automatic summary, we obtain a score of precision. The two scores can be combined to obtain an *F*-score for the automatic summary. Broadly, the recall depicts how much relevant information we obtained from the manual summary, and the precision gives a picture of how much relevant information we have in the automatic summary. ROUGE scores are between 0 and 1, where 1 is better.

From the variety of ROUGE methods, we make use of ROUGE-1, ROUGE-2 and ROUGE-SU4. To have a better idea of how ROUGE calculates the scores, we present the equations and some situations with fabricated examples. Let AS be an automatic summary, be MS a manual summary, and be 2-GRAM (AS, MS) the number of bigrams (2-grams) co-occurring in both summaries. Then, we can calculate the scores for ROUGE-2 as follows (Equation 10):

(10) $$\begin{array}{c} \text{Recall}=\frac{2-\text{GRAM}(MS,\;AS)}{m},\\ \text{Precision}=\frac{2-\text{GRAM}(MS,\;AS)}{n},\\ F-\text{score}=\frac{2\text{RecallPrecision}}{\text{Recall}+\text{Precision}}, \end{array}$$

where *m* stands for the number of *n*-grams in MS and *n* stands for the number of *n*-grams in AS. Now, imagine a manual summary (*MS*) and an automatic summary (*AS*) that we want to evaluate:

MS: ArgR has N-terminal and C-terminal domains

*AS*: *ArgR has N-terminal domain*

We can see that the automatic summary not only includes part of the relevant information of the manual summary but also words and a word order that is very similar. Then, given the following bigrams in each summary, there are two overlaps: ‘ArgR has’ and ‘has N-terminal’. The ROUGE-2 scores for *AS* are then recall* *= 2/5 = 0.4, precision = 2/3 = 0.6, and *F*-score = 0.5.

*MS:* (‘**ArgR has**’, ‘**has N-terminal**’, ‘N-terminal and’, ‘and C-terminal’, ‘C-terminal domains’); *m = *5

*AS:* (‘**ArgR has**’, ‘**has N-terminal**’, ‘N-terminal domain’); *n = *3

On the one hand, the recall of 0.4 shows us that the automatic summary carries less than the half the relevant information from the manual summary. On the other hand, the precision of 0.6 shows that more than the half of the automatic summary is relevant information. Now, what happens if we evaluate a summary with the same information but that is presented in a different word order? Imagine the next automatic summary, AS’, which includes almost all the words of *AS* but in the opposite order. In this case, there are no overlapping bigrams, and ROUGE-2 assigns a zero score to this automatic summary, despite it containing all the relevant information.

*AS’*: *domain N-terminal of ArgR*

*AS’* bigrams*:* (‘domain N-terminal’, ‘N-terminal of’, ‘of ArgR’)

Taking into consideration the previous situation, we also used ROUGE-SU4 to evaluate the automatic summaries. ROUGE-SU4 measures the cooccurrence of unigrams together with skip-bigrams of distance 4, that is, all pairs of words separated at most by four words (following the sentence order). Let SKIP4(MS, AS) be the overlapped unigrams and skip-bigrams; then we can calculate ROUGE-SU4 scores as shown in Equation (11):

(11) $$\begin{array}{c} \text{Recall}=\frac{SKIP4(MS,\;AS)}{m},\\ \text{Precision}=\frac{SKIP4(MS,\;AS)}{n}. \end{array}$$

Then, based on the following unigrams and skip-bigrams of the manual summary, *MS*, and the automatic summary, *AS’*, we obtained ROUGE-SU4 recall = 2/20 = 0.1, precision = 2/10 = 0.2, and *F*-score = 0.1, because there are only two coinciding unigrams. This score is low, but it takes into consideration isolated words, and the effect of word order is reduced.

*MS:* (‘**ArgR**‘, ‘has’, ‘**N-terminal**‘, ‘and’, ‘C-terminal ‘, ‘domains’) + (‘ArgR has’, ‘ArgR N-terminal’, ‘ArgR and’, ‘ArgR C-terminal’, ‘has N-terminal’, ‘has and’, ‘has C-terminal’, ‘has domains’, ‘N-terminal and’, ‘N-terminal C-terminal’, ‘N-terminal domains’, ‘and C-terminal’, ‘and domains’, ‘C-terminal domains’); *m *= 20.

*AS’*: (‘domain’, ‘**N-terminal**’, ‘of’, ‘**ArgR**’,) + (‘domain N-terminal’, ‘domain of’, ‘domain ArgR’, ‘N-terminal of’, ‘N-terminal ArgR’, ‘of ArgR’); *n *= 10.

In addition, ROUGE can be calculated by eliminating stop words (highly frequent words, prepositions, conjunctions and determiners). Therefore, for overlapped unigrams, we would have a better idea of how much relevant information we retrieved, since the comparison would put aside function words. However, overlapped bigrams become more difficult to find, because stop words commonly appear in the middle of words.

As we already mentioned, we created our automatic summaries by the concatenation of the automatically classified sentences. This simple approach brings some problems of redundancy and an excess of irrelevant information; indeed, it is anticipated that our summaries would be very long compared with the manual summaries. Nevertheless, we will tackle this problem in future work, because it is not trivial to either merge similar sentences or to filter out irrelevant parts of sentences. It is another complete NLP task to transform an extractive summary into a more compact version. In addition, the idea of irrelevant information in the automatic summary is relative, because some of that information could be valuable for the curation work. In addition, the length of the automatic summary is also relative, because with this method we are summarizing a collection of complete scientific articles.

With these automatic summaries, we can expect that ROUGE *F*-scores would not reveal how much relevant information they have. The cause is the low precision because, as we explained before, the number of overlapped *n*-grams is divided by the total *n*-grams of the automatic summary, which will be a high number. Looking at the recall would be a better way to realize how much relevant data about TFs we are summarizing. This is because the overlapped n-grams are divided by the expected n-grams of the manual summary. In other words, precision measures how much information in the automatic summary is relevant, but, in the first steps of our research, we are more interested in measuring how much relevant information from the manual summaries is also in the automatic summaries.

To prove this, we ran ROUGE 1.5.5 to evaluate a fabricated automatic summary, AS’’, against the manual summary *MS* (see Supplementary Table S18)*.* The first sentence in the *AS’’* has the same information and word order as the *MS*. The second sentence has the same information but it appears in an inverted order. Last, the third sentence contains additional information. Then, the automatic summary is very long compared with the manual one. For all ROUGE scores, the recall was very high while the precision was very low. As a consequence, the *F*-score of the automatic summary was also low. This confirms that the recall better shows that most of the important information of the manual summary is in the automatic one, especially with ROUGE-1.

## Results

### Automatic classification

Comparing the *F*-score of the two strategies for model selection, the strategy with the validation dataset surpassed the strategy with only cross-validation (Table 7). In both cases, the best model was an SVM using lemmas, term tags, and frequent-word tags as features to describe sentences. The training with sentences instead of fragments was better. The hyperparameters of these models are shown in Supplementary Table S19.

**Table 7.** Descriptions of the two selected classifiers

Strategy	Classifier	Features	*n*-grams	Remove stop words?	SVD	**Comp**^a^	Values	*F*-score
Validation dataset	SVM	Lemmas, term tags, and frequent-word tags	1 + 2	No	Yes	200	TF-IDF	0.888
Only cross-validation	SVM	Lemmas, term tags, and frequent-word tags	1 + 2	No	No		Binary	0.867

a Componentes.

To decide on the best model for automatic summarization, we assessed the performance of the two models with the test dataset. Whereas the averaged performance for the five TFs decreased for the model selected with the validation dataset (*F*-score, 0.8470), it remained for the model selected with only cross-validation (*F*-score, 0.8689). It seems that the second model generalized better than the first one. Hence, we decided to utilize this model. We will describe its performance in following paragraphs.

Because the class OTHER was overrepresented in the datasets, it was not surprising that this class was the best classified for all TFs (Table 8). Concerning the classes DOM and RP, although the *F*-scores for these two classes were similar, the model performed better for sentences about structural domains than for sentences about regulated biological processes. For example, this model could not classify any sentence of class RP for ArgR and GntR.

**Table 8.** Performance of the best classifier for TF and class

TF	DOM			OTHER			RP		
	Precision	Recall	*F*-score	Precision	Recall	*F*-score	Precision	Recall	*F*-score
ArgR	1	0.12	0.21	0.86	1	0.92	0	0	0
CytR	1	1	1	1	1	1	1	1	1
FhlA	1	0.25	0.4	0.89	1	0.94	—	—	—
GntR	0.67	0.25	0.36	0.95	0.98	0.97	0	0	0
MarA	0.77	0.26	0.39	0.78	0.97	0.87	1	0.67	0.8
Average^a^	**0.89**	0.38	0.47	0.9	**0.99**	0.94	**0.5**	0.42	0.45

a The highest score between averaged precision and recall is shown in boldface.

We also observed that this model attained higher precision than recall for the classes DOM and RP. This means that the sentences classified as DOM and RP were frequently true, but many sentences of these classes were left aside. For example, from the three sentences classified as DOM for ArgR, the three were true but the model could not classify correctly the remaining 22 (see the confusion matrices of all TFs in Supplementary Table S20). In contrast, the recall for the class OTHER was better than the precision, that is, almost all the sentences about other kinds of information in the dataset were classified correctly, but a few were confused with another class. For example, three sentences of class OTHER were classified as DOM for MarA. The lowest score of class DOM was for ArgR, and the best score was for CytR. In fact, CytR had a perfect classification for all classes. For class RP, the lowest scores were for ArgR and GntR.

### Automatic summarization

As we expected, the ROUGE recall score was higher than the precision score (Table 9). The best recall was for CytR and MarA, but ArgR had a better balance of precision and recall. Only the shortest summaries (ArgR and GntR) obtained better precision scores. Generally, we can say that automatic summaries had between 40 and the 80% of the words of the manual summary (see recall in Table 9). In addition, to have an idea of whether these words were relevant data instead of general words, ROUGE-1 without stop words was used for an approximation. In this case, automatic summaries covered between 30 and 70% of the data of the manual summary.

**Table 9.** Evaluation of automatic summaries with ROUGE-1 with and without stop words

TF	ROUGE-1						
	With stop words			Without stop words			Summary (words)
	Recall	Precision	*F*-score	Recall	Precision	*F*-score	
ArgR	0.553	**0.442**	**0.491**	0.463	**0.348**	**0.397**	154
CytR	**0.86**	0.087	0.158	0.759	0.066	0.121	1,124
FhlA	0.753	0.109	0.19	0.63	0.091	0.159	676
GntR	0.418	0.241	0.306	0.326	0.182	0.233	137
MarA	0.821	0.063	0.117	**0.761**	0.05	0.094	1,103
Average	0.681	0.188	0.252	0.587	0.147	0.201	

The best scores are shown in boldface.

ROUGE-SU4 entails a more severe evaluation method. It takes into consideration skip-bigrams of distance 4 and unigrams. Again, summaries of CytR and MarA had the best recall, and ArgR had the best *F*-score (Table 10). All ROUGE scores are shown in Supplementary Table S21. Concerning unigrams and skip-bigrams, our initial automatic summaries had up to 40 and 30% of the information of the manual summaries, with and without stop words, respectively.

**Table 10.** Evaluation of automatic summaries with ROUGE-SU4 with and without stop words

TF	ROUGE-SU4			
	With stop words		Without stop words	
	Recall	*F*-score	Recall	*F*-score
ArgR	0.277	**0.245**	0.223	**0.19**
CytR	0.428	0.077	0.361	0.056
FhlA	0.392	0.097	0.269	0.066
GntR	0.146	0.106	0.095	0.067
MarA	**0.445**	0.062	**0.362**	0.042

Best scores in bold face.

## Discussion

We can confirm that the strategy of using sentences from manual summaries to classify sentences of articles was challenging. The results of the model assessment revealed that the model could classify correctly only a few of sentences from the articles (low recall), but the classification was regularly correct (high precision). A disadvantage of this model is that it puts aside several sentences which could be valuable for curators. Nevertheless, the sentences from articles correctly classified, although they were few in number, can give curators a significant part of the relevant information they need, because they were obtained by a model trained with the sentences of manual summaries, which condense a great deal of relevant information. Moreover, the perfect classification of the sentences of both classes for CytR revealed that our strategy could obtain good results.

**Figure 6.**
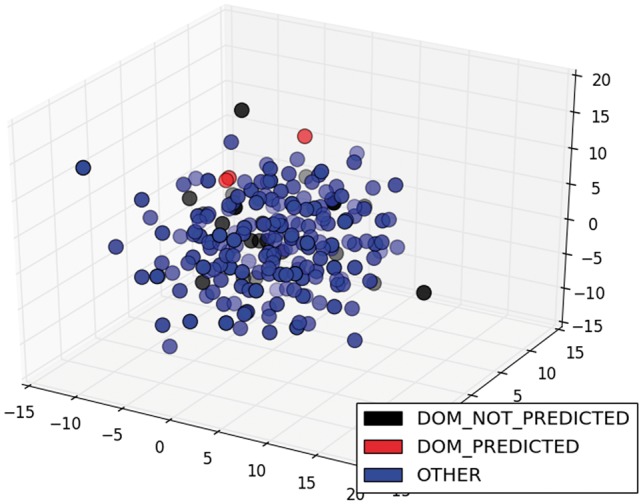
MDS of sentences of the class DOM for ArgR.

**Figure 7.**
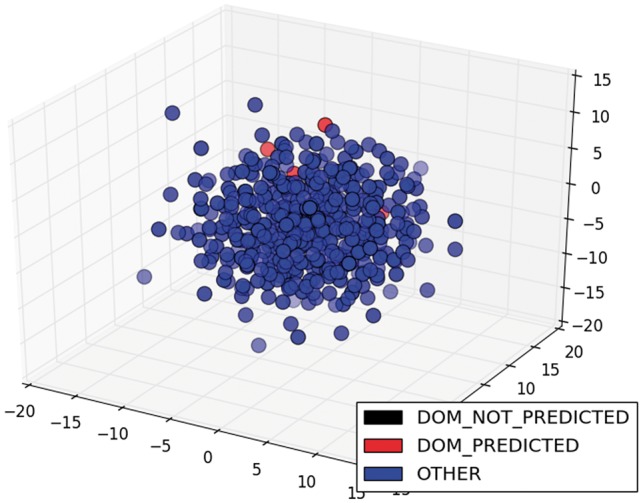
MDS of sentences of the class DOM for CytR.

The low scores in classification and its variation among different TFs were due to the diversity of ways to write sentences about the information we needed. The greater the variety of ways, the lower the performance of the classifier, because it was trained with restricted examples. For example, the model only classified correctly 3 of the 25 sentences of the class DOM of ArgR. If we were to graph the sentence vectors about this TF in three dimensions (3D), we would see that the predicted sentences were relatively close and two of them were very similar (red circles in Figure 6), while the unpredicted sentences of this class were sparser (black circles in Figure 6). On the other hand, the classification of the same class (DOM) for CytR was perfect, because the four sentences were relatively close and less sparse (red circles in Figure 7). To graph these results in 3D, we used multidimensional scaling (MDS), which is a widely used technique to visualize in low dimensionality the structure of data (35). The color degradation gives the appearance of depth.

**Figure 8.**
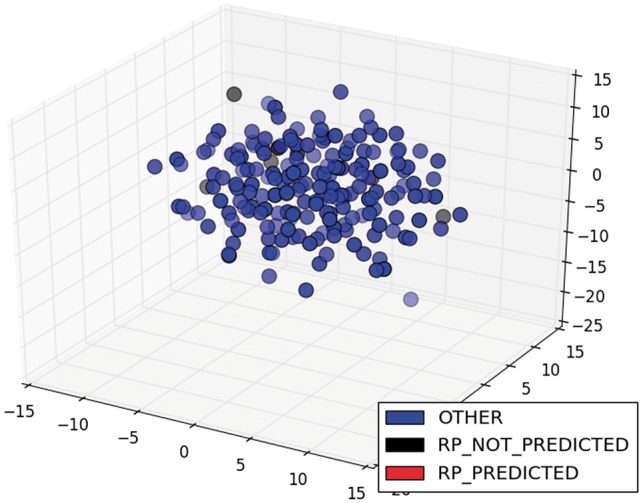
MDS of sentence of the class RP for ArgR.

**Figure 9.**
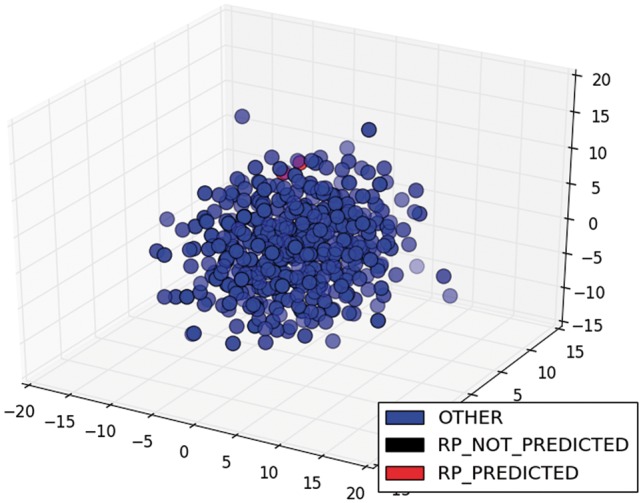
MDS of sentence of the class RP for CytR.

In addition, the model could not classify any of the nine sentences of the class RP for ArgR. In this case, these sentences were also sparser (black circles in Figure 8) than the two sentences of the same class for CytR, which were predicted correctly (red circles in Figure 9). Clearly, this is a limitation of our proposal, and it comes from the selected training strategy, which we can improve by incorporating new classified sentences of articles into the training dataset to improve the classifier.

On the other hand, our empirical results revealed a combination of aspects to be considered for future work. First, use of training sentences was a better strategy than use of training fragments. This is because the sentences had more context. Also, adding term tags and frequent-word tags to lexical items (lemmas) helped us obtain the required sentences. This result makes sense, because some words chosen by the curators to write the summaries should be different from words employed by the authors; therefore, the tags can give more general and standardized information to the classifier. Moreover, the combination of unigrams and bigrams was a good option. Last, we observed that a *K*-fold-stratified cross-validation strategy was suitable for our problem with scarce positive examples. All these findings will be taken into consideration to eventually generate summaries with more properties about TFs.

We will consider some strategies to tackle the problem of low classification performance. First, we will explore semantic representations of sentences. This could give classifiers information about the semantic similarities of sentences instead of word similarity. We expect that the classifiers recognize similar sentences despite the way they are written. Another strategy will be to test two-class classification instead of multiclass, which was the strategy employed in this study. Likely, a classifier could discriminate better between two classes than three. Also, cases where a sentence belongs to more than one class could be considered with this strategy. An additional approach would be to employ more sophisticated strategies to deal with imbalanced datasets, such as those that might results from over- or undersampling. Finally, the potential benefits of testing more classifiers will be taken into account.

Despite the results in our sentence classifications, our automatic summaries comprised part of the relevant information recorded by the curators in the manual summaries. This was confirmed by recall, which is a more suitable score to indicate how much relevant information our automatic summaries had, given the simple approach to summarize by concatenating sentences. According to this score, the summaries of CytR and MarA covered up to 70% of the relevant data from the manual summary. The low precision was because the automatic summaries were too long compared with the manual summaries. In the long run, we will tackle this problem by testing some strategies, such as clustering of sentences, sentence compression, and sentence fusion.

To have a clearer idea of how much information the automatic summaries share with the manual ones, we made some comparisons using the worst and the best summary, according to recall score. The worst summary was for GntR (0.146 of ROUGE-SU4 recall), which encompassed only five relevant data elements related to structural domain and regulated biological processes from the manual summary (Supplementary Table S22). The low recall of this summary was due to many terms that did not coincide with terms in the manual summary; however, the automatic summary held relevant information for curation. In classification, GntR also obtained the lowest scores (Table 8).

The best summary was for MarA (ROUGE-SU4 recall of 0.445 and 0.362 with and without stop words, respectively). The *F*-score of this summary was very low due to it having much more information (Supplementary Table S23). Despite the low *F*-score, this summary shared all relevant data with the manual summary. Classification of sentences of this TF was not the best (Table 8), but it was the second best.

The best automatic summary according to the ROUGE *F*-score was for ArgR (Supplementary Table S24). This summary included almost all of the relevant data of the manual summary with a great level of coincidence of words and briefness. It is encouraging that our strategy obtained this level of useful summaries. Nevertheless, future analysis working with an open collection will be necessary, because we used articles already reviewed by our curators for writing the manual summaries.

The empirical results showed that training an automated model by using manual summaries to classify sentences of articles is a valid strategy, although it requires improvements, because the classification score was low. We can assume that there are sentences of articles which resemble sentences of manual summaries of RegulonDB, which are feasible to classify. By using manual summaries as training data, we reduced the human effort to create a dataset. Now, with this classifier, we can obtain sentences of articles to enrich the training examples. Concerning the automatic summaries, despite the classification performance, the results of our summary evaluation confirmed that we can generate summaries that contain relevant information of the manual summaries.

An important achievement of our research is that we established an initial strategy to cover more properties about TFs. Our plan is to continue with properties which have a controlled vocabulary, such as: (a) the meaning of the TF acronym, indicating whether it is a repressor, activator, or dual transcriptional regulator; (b) the active and inactive conformations of the TF; (c) information about the TFBS features (such as size, and symmetry of the consensus sequence); (d) information about evolutionary features of the TF; and (e) information about the organization of the transcription unit that contains the gene encoding the TF. The first step will be to apply the same strategy, that is, extracting training examples from manual summaries, collecting term lists and tagging sentences. If the results of this strategy were to be applied for other remaining properties of the TFs, these automatic summaries could be useful for curators to write summaries about TFs of RegulonDB that do not currently have one. Nevertheless, increasing the number of properties will make the classification task more challenging, and so we must improve the classification performance first.

## Conclusions

In this article, we have presented a proposal to generate initial extractive, multidocument, automatic summaries about two properties of TFs: the structural domains constituting the TF and the biological processes in which the regulated genes are involved. The summarization is accomplished by automatic classification of sentences from scientific articles about *E. coli* by means of an SVM classifier. Then, these sentences are concatenated to create summaries. The evaluation of these initial automatic summaries indicated that the strategy is valid. These summaries contain part of the relevant information that curators of RegulonDB have included in the manual summaries. Yet, some improvement is required.

This strategy will be expanded to summarize more properties of TFs. Then, we will be able to generate suggested sentences with relevant information to help the curation work without any loss of quality. Eventually, we will consider applying this strategy to new article collections for other organisms.

## Supplementary data

Supplementary data are available at *Database* Online.

## Supplementary Material

Supplementary Data
