# Supplementary material for: First steps in automatic summarization of transcription factor properties for RegulonDB: classification of sentences about structural domains and regulated processes
Source: Database (Oxford). 2017 Sep 26;2017:bax070. doi: 10.1093/database/bax070 (PMC5737074; doi:10.1093/database/bax070)
Supplement: Supplementary Data [file bax070_supp_material.doc]

**First steps in automatic summarization of transcription factor properties for RegulonDB: classification of sentences about structural domains and regulated processes**

Carlos-Francisco Méndez-Cruz^1,*^, Socorro Gama-Castro^1^, Citlalli Mejía-Almonte^1^, Marco-Polo Castillo-Villalba^1^, Luis-José Muñiz-Rascado^1^, and Julio Collado-Vides^1^

^1^ Computational Genomics Program, Center for Genomic Sciences, National Autonomous University of Mexico, Cuernavaca, Morelos, Postcode: 62100, Mexico. Av. Universidad s/n, Colonia Chamilpa.

* Corresponding author: Tel: +52 777 3132063; Fax: +52 7773175581; Email: [cmendezc@ccg.unam.mx](mailto:collado@ccg.unam.mx)

# Supplementary material

## Tables

| Table 11. Nomenclature | |
| --- | --- |
| **Term** | **Definition** |
| Bigram | Two consecutive words, lemmas, or tags (features) from a sentence representation. The bigrams of the sentence “SutR is a small transcription factor” are ('SutR is', 'is a', 'a small', 'small transcription', 'transcription factor'). |
| Collocation | In linguistics, a collocation is a set of words which commonly appear together, such as winding road or make progress. |
| *F* | ROUGE *F*-score = 2RP/R + P |
| Frequent-word tag | Tags assigned to frequent words in sentences from manual summaries. If a word of a sentence appears in the 100 most frequent words in sentences about structural domains, we assigned the tag FWDOM. If a word of a sentence appears in the 100 most frequent words in sentences about regulated processes, we assigned the tag FWRP. |
| *F*-score | The harmonic mean of precision and recall |
| *K*-fold cross-validation | The *K*-fold cross-validation method splits training data into *K* parts and uses (*K* – 1) parts to train and the remaining part for validation. It trains *K* times, always validating with a different part of the data. |
| Lemma | A normalized or canonical representation of a set of morphologically related words. The lemma of “motif” and “motifs” is “motif.” The lemma of “activates.” “activated,” and “activating” is “activate.” |
| Lemmatization | The process of assigning lemmas to words of sentences. |
| NB | Naïve Bayes classifier (Bernoulli, Gaussian, or multinomial) |
| ROUGE precision | The overlapped n-grams over the total n-grams of the automatic summary |
| Part-of-speech (POS) | Lexical classes and grammatical categories of words, such as “Verb 3er-Person Present” for “runs,” or “Noun Plural” for “tables.” |
| Part-of-speech tag (POS tag) | Tag representing the part-of-speech (POS) of a word. For example, the POS of the verb “likes” is “Verb, 3rd person singular present”; this POS is represented by the tag VBZ in the Penn Treebank tag set. |
| Part-of-speech tagging (POS tagging) | The process of assigning part-of-speech tags to words of sentences. |
| Precision | Ratio of how many examples predicted as positive are true; Precision = TP/(TP + FP) |
| ROUGE recall | The overlapped n-grams over the total n-grams of the manual summary |
| Recall | Ratio of how many positive examples are predicted from all positive examples; Recall = TP/(TP + FN) |
| ROUGE | A widely used method for summary evaluation based on the number of n-grams of the automatic summary that cooccur in the total set of expected n-grams of the manual summary. |
| ROUGE-1, ROUGE-2, and ROUGE-SU4 | ROUGE-1 and ROUGE-2 weight the cooccurrence of unigrams and contiguous bigrams, respectively. ROUGE-SU4 measures the cooccurrence of unigrams together with skip-bigrams of distance 4, that is, all pairs of words separated at most by four words following the sentence order. |
| Singular Value Decomposition (SVD) | A kind of matrix decomposition used to get a low-rank approximation. |
| Stop words | The words to be removed from text data; generally they are function words or highly frequent words. |
| SVM | Support Vector Machine classifier |
| Tag | Descriptive label assigned to a word or term, for example, DPOS for domain positions, PRO for biological processes, NOUN for table, VERB for run |
| Tag set | Set of POS tags; generally, a tag set is taken from a well-known corpus, for example, the Penn Treebank tag set |
| Term tag | A term tag was assigned if the word appeared in one of the term lists that we gathered, e.g., transcription factors, biological processes, molecular functions, domain positions, domain families, and domain structural motifs. Possible tags were TF, PRO, MF, DPOS, DFAM, and DMOT, respectively. |
| Test dataset | Set of manually classified sentences from articles about the five TFs used to estimate the general performance of the best classification model |
| *tf-idf* weight | The *tf-idf* weight gives more importance to words that better describe a document. The weight is very low for terms (i.e., words) that occur in most of the examples of a dataset, and it is higher for terms occurring in only some of them. Then, let *tf(j)* be the term frequency of *j* and *idf(j)* the inverse document frequency of *j*; the *tf-idf* weight of *j* is *tf-idf(j) = tf(j) x idf(j)*, and *idf(j) = log(N/df(j)),* where *N* stands for the number of examples in the dataset and *df(j)* stands for the number of examples that contain the term *j.* |
| Training dataset | Set of manually classified sentences used to fit the classification model and learn decision criteria |
| Trigram | Three consecutive words, lemmas, or tags (features) from a sentence representation. The trigrams of the sentence “SutR is a small transcription factor” are ('SutR is a', 'is a small', 'a small transcription', 'small transcription factor') |
| Unigram | One word, lemma, or tag (feature) from a sentence representation; the unigram of the sentence “SutR is a small transcription factor” are ('SutR', 'is', 'a', 'small', 'transcription', 'factor') |
| Validation dataset | Set of manually classified sentences used to select the best classification model among fitted models |
| Vector Space Model (VSM) | In this model, sentences are represented as vectors where its components correspond to features, for example, words. First, the set of features from all sentences is obtained (usually called vocabulary), and then the frequency of the word in the sentence is used as the value of the component. |
| Vectorization | The process to convert text data into vectors |

| **Table 12. Confusion matrix for a classification problem of two classes** | | |
| --- | --- | --- |
| **True class** | **Predicted class** | |
|  | Positive | Negative |
| Positive | True positive (TP) | False negative (FN) |
| Negative | False positive (FP) | True negative (TN) |

| **Table 13. Example of prediction and evaluation of two classifiers** | | |
| --- | --- | --- |
| **True classes** | **Predicted classes** | |
|  | **Classifier 1** | **Classifier 2** |
| DOM | DOM | DOM |
| DOM | OTHER | DOM |
| DOM | OTHER | DOM |
| OTHER | OTHER | DOM |
| OTHER | OTHER | DOM |
| OTHER | OTHER | DOM |
|  |  |  |
| **Classifier 1** | | |
| **True class** | **Predicted class** | |
|  | Positive | Negative |
| Positive | 1 | 2 |
| Negative | 0 | 3 |
| **Precision** | **Recall** | ***F*-Score** |
| 1.000 | 0.333 | 0.500 |
|  |  |  |
| **Classifier 2** | | |
| **True class** | **Predicted class** | |
|  | Positive | Negative |
| Positive | 3 | 0 |
| Negative | 3 | 0 |
| **Precision** | **Recall** | ***F*-Score** |
| 0.500 | 1.000 | 0.667 |

| **Table 14. Five randomly selected examples of sentences of each class** |  |
| --- | --- |
| **Sentence** | **Class** |
| ArgP, which belongs to the LysR-family, has a helix-turn-helix motif located close to the N-terminus. | DOM |
| CsgD belongs to the FixJ/LuxR/UhpA-family, which is characterized by a C-terminal domain that contains a potential helix-turn-helix DNA-binding motif and a receiver-domain in the N-terminal region. | DOM |
| The L1 loop is known to interact with σ^54^ , presumably with region I of σ^54^. | DOM |
| SutR is a small transcription factor with a helix-turn-helix ( HTH ) motif and belongs to the Cro-C1-type superfamily. | DOM |
| The monomer of this transcription factor contains two domains: the N-terminal domain is responsible for binding DNA and dimerization and also contains a winged-helix-turn-helix motif. | DOM |
| Although HipA does not bind the Hip regulatory region, it plays an indirect role via its binding to HipB. | OTHER |
| CusR belongs to the two-component system CusS/CusR, which responds to increases in the copper concentration. | OTHER |
| It has been suggested that ZraS functions as a zinc receptor. | OTHER |
| CsiR is expressed upon carbon starvation and has a repressor effect during the stationary phase. | OTHER |
| YiaJ is negatively autoregulated and coordinately represses transcription of the divergent yiaK-S operon, related to degradation of l-lyxose . | OTHER |
| YdeO activates genes involved in the cellular response to acid resistance. | RP |
| Based on analysis with the Genomic SELEX screening system, PgrR was identified as a repressor of the expression of genes of the initial enzymes for peptidoglycan ( PG ) peptide-degradation as well as genes of the switch control between recycling and degradation of PG peptides, which are induced upon exposure to heat shock or membrane distortion. | RP |
| CRP activates a gene potentially related to persistence. | RP |
| It is also involved in the bacterial stringent-response. | RP |
| MarA, multiple antibiotic resistance, participates in controlling several genes involved in resistance to antibiotics, oxidative stress, organic solvents, and heavy metals. | RP |

| Table 15. The 15 most frequent words in training sentences | | |
| --- | --- | --- |
| **Structural domains** |  | **Regulated process** |
| domain |  | genes |
| N-terminal |  | CRP |
| C-terminal |  | transport |
| helix-turn-helix |  | involved |
| DNA-binding |  | DNA |
| contains |  | transcription |
| two |  | metabolism |
| motif |  | acid |
| domains |  | catabolism |
| binding |  | resistance |
| DNA |  | expression |
| protein |  | operon |
| involved |  | also |
| family |  | synthesis |
| region |  | cell |

| Table 16. The tag set for biological terms | |
| --- | --- |
| **Biological term** | **Tag** |
| Transcription factor | TF |
| Biological process | PRO |
| Molecular function | MF |
| Domain position | DPOS |
| Domain family | DFAM |
| Domain structural motif | DMOT |

| **Table 17. Grid of hyperparameters to optimize** | | | |
| --- | --- | --- | --- |
| **Classifier** | **Kernel** | **Kernel Hyperparameters** | **Hyperparameters** |
| SVM | linear | --- | C: expon(scale=100), class_weight: ['balanced', None] |
| SVM | radial basis function (rbf) | gamma: expon(scale=.1) | C: expon(scale=100), class_weight: ['balanced', None] |
| SVM | polynomial (poly) | gamma: expon(scale=.1), degree: [2, 3] | C: expon(scale=100), class_weight: ['balanced', None] |
| Multinomial NB | --- | alpha: expon(scale=1.0) |  |
| Bernoulli NB | --- | alpha: expon(scale=1.0) |  |

| **Table 18. ROUGE scores for a long automatic summary** | | | | |
| --- | --- | --- | --- | --- |
| MS*: ArgR has N-terminal and C-terminal domains* | | | | |
| AS’’*: ArgR has two domains : the N-terminal domain and the C-terminal domain. C-terminal and N-terminal domains belong to ArgR. Based on cross-linking analysis of wild-type and mutant ArgR proteins, it has been shown that the C-terminus is more important in cer/Xer site-specific recombination than in DNA binding* | | | | |
|  | ***Recall*** | ***Precision*** | ***F-score*** |  |
| ROUGE-1 | 1.0000 | 0.1455 | 0.2540 |  |
| ROUGE-2 | 0.7143 | 0.0926 | 0.1639 |  |
| ROUGE-SU4 | 0.8438 | 0.0860 | 0.1561 |  |

| **Table 19. Hyperparameters of the two selected classifiers** | | | | | |
| --- | --- | --- | --- | --- | --- |
| **Classifier** | **Kernel** | **Degree** | **Gamma** | **C** | **class_weight** |
| SVM | Polynomial | 2 | 0.324 | 220.63 | None |
| SVM | Linear | --- | --- | 0.030 | None |

| **Table 20. Confusion matrices of the five TFs** | | | | | | | | |
| --- | --- | --- | --- | --- | --- | --- | --- | --- |
| **ArgR** |  |  |  |  |  |  |  |  |
|  |  | **Predicted classes** | | |  |  |  |  |
|  |  | **DOM** | **OTHER** | **RP** | **Sentences** | **Precision** | **Recall** | ***F*-score** |
| **True classes** | **DOM** | 3 | 21 | 1 | 25 | 1.00 | 0.12 | 0.21 |
|  | **OTHER** | 0 | 182 | 0 | 182 | 0.86 | 1.00 | 0.92 |
|  | **RP** | 0 | 9 | 0 | 9 | 0.00 | 0.00 | 0.00 |
|  |  |  |  |  |  |  |  |  |
| **CytR** |  |  |  |  |  |  |  |  |
|  |  | **Predicted classes** | | |  |  |  |  |
|  |  | **DOM** | **OTHER** | **RP** | **Sentences** | **Precision** | **Recall** | ***F*-score** |
| **True classes** | **DOM** | 4 | 0 | 0 | 4 | 1.00 | 1.00 | 1.00 |
|  | **OTHER** | 0 | 425 | 0 | 425 | 1.00 | 1.00 | 1.00 |
|  | **RP** | 0 | 0 | 2 | 2 | 1.00 | 1.00 | 1.00 |
|  |  |  |  |  |  |  |  |  |
| **FhlA** |  |  |  |  |  |  |  |  |
|  |  | **Predicted classes** | |  |  |  |  |  |
|  |  | **DOM** | **OTHER** |  | **Sentences** | **Precision** | **Recall** | ***F*-score** |
| **True classes** | **DOM** | 1 | 3 |  | 4 | 1.00 | 0.25 | 0.40 |
|  | **OTHER** | 0 | 25 |  | 25 | 0.89 | 1.00 | 0.94 |
|  |  |  |  |  |  |  |  |  |
| **GntR** |  |  |  |  |  |  |  |  |
|  |  | **Predicted classes** | | |  |  |  |  |
|  |  | **DOM** | **OTHER** | **RP** | **Sentences** | **Precision** | **Recall** | ***F*-score** |
| **True classes** | **DOM** | 2 | 6 | 0 | 8 | 0.67 | 0.25 | 0.36 |
|  | **OTHER** | 1 | 180 | 2 | 183 | 0.95 | 0.98 | 0.97 |
|  | **RP** | 0 | 3 | 0 | 3 | 0.00 | 0.00 | 0.00 |
|  |  |  |  |  |  |  |  |  |
| **MarA** |  |  |  |  |  |  |  |  |
|  |  | **Predicted classes** | | |  |  |  |  |
|  |  | **DOM** | **OTHER** | **RP** | **Sentences** | **Precision** | **Recall** | ***F*-score** |
| **True classes** | **DOM** | 10 | 28 | 0 | 38 | 0.77 | 0.26 | 0.39 |
|  | **OTHER** | 3 | 105 | 0 | 108 | 0.78 | 0.97 | 0.87 |
|  | **RP** | 0 | 1 | 2 | 3 | 1.00 | 0.67 | 0.80 |

| **Table 21. ROUGE evaluation of automatic summaries with and without stop words** | | | | | | | | | |
| --- | --- | --- | --- | --- | --- | --- | --- | --- | --- |
| **With stop words** | | | | | | | | | |
|  | **ROUGE-1** | |  | **ROUGE-2** | |  | **ROUGE-SU4** | |  |
| **TF** | **R** | **P** | **F** | **R** | **P** | **F** | **R** | **P** | **F** |
| ArgR | 0.553 | 0.442 | 0.491 | 0.238 | 0.190 | 0.211 | 0.277 | 0.220 | 0.245 |
| CytR | 0.860 | 0.087 | 0.158 | 0.372 | 0.037 | 0.068 | 0.428 | 0.043 | 0.077 |
| FhlA | 0.753 | 0.109 | 0.190 | 0.354 | 0.051 | 0.089 | 0.392 | 0.055 | 0.097 |
| GntR | 0.418 | 0.241 | 0.306 | 0.103 | 0.059 | 0.075 | 0.146 | 0.083 | 0.106 |
| MarA | 0.821 | 0.063 | 0.117 | 0.422 | 0.032 | 0.059 | 0.445 | 0.033 | 0.062 |
| R =recall, P = precision, F = *F*-score | | | | | |  |  |  |  |
|  |  |  |  |  |  |  |  |  |  |
| **Without stop words** | | | | | | | | | |
|  | **ROUGE-1** | |  | **ROUGE-2** | |  | **ROUGE-SU4** | |  |
| **TF** | **R** | **P** | **F** | **R** | **P** | **F** | **R** | **P** | **F** |
| ArgR | 0.463 | 0.348 | 0.397 | 0.167 | 0.125 | 0.143 | 0.223 | 0.166 | 0.190 |
| CytR | 0.759 | 0.066 | 0.121 | 0.281 | 0.024 | 0.044 | 0.361 | 0.030 | 0.056 |
| FhlA | 0.630 | 0.091 | 0.159 | 0.283 | 0.040 | 0.071 | 0.269 | 0.037 | 0.066 |
| GntR | 0.326 | 0.182 | 0.233 | 0.095 | 0.053 | 0.068 | 0.095 | 0.052 | 0.067 |
| MarA | 0.761 | 0.050 | 0.094 | 0.311 | 0.020 | 0.038 | 0.362 | 0.023 | 0.042 |
| R =recall, P = precision, F = *F*-score | | | | | |  |  |  |  |

| **Table 22. Comparison of the manual and automatic summary of GntR** |
| --- |
| **Manual summary** |
| Accordingly , this transcriptional repressor family protein is composed of two domains : a conserved N-terminal domain which contains the DNA-binding region , and the carboxy-terminal domain , which is involved in effector-binding and oligomerization .  The Gluconate repressor , GntR , is a transcription factor that negatively regulates the operon involved in the catabolism of d-gluconate via the Entner-Doudoroff pathway and also represses genes involved in two different systems related to d-gluconate uptake : gluconate I and gluconate II . |
| **Automatic summary** |
| As shown in Fig. 3 , the HTH of GntH has quite high similarity to that of GntR compared with those of other members .  structure with those of the repressors in the CytR family revealed that GntR also has a helix-turnhelix motif at its N-terminus , which may be responsible for DNA-binding and it may also have domains related to dimerization and inducer-binding at its C-terminus ( Weickert & Adhya , 1992 ) .  GntR was also genetically shown to be involved in the control of expression of gntT and of some genes participating in FITC , fluorescein isothiocyanate ; gntK , gene encoding gluconate the Entner -- Doudoroff pathway ( Nagel de Zwaig et al. , kinase in GntI ; GntK , gene product of gntK ; GntI , major gluconate 1973 ; Zwaig et al. , 1973 ; Egan et al. , 1992 ) . |

| **Table 23. Comparison of the manual and automatic summary of MarA** |
| --- |
| **Manual summary** |
| These three proteins belong to the AraC/XylS-family of transcriptional regulators and as with other members of this family they have two helix-turn-helix ( HTH ) motifs for DNA-binding , one of them , located in the N-terminal region , interacts with the element RE1 of the mar box , and the HTH located in the C-terminal region interacts with the element RE2 .  MarA, multiple antibiotic resistance, participates in controlling several genes involved in resistance to antibiotics , oxidative stress, organic solvents and heavy metals . |
| **Automatic summary** |
| […]  The SoxS and MarA proteins consist only of the conserved DNA-binding domain ( ∼ 100 residues ) that is present in all AraC/XylS-family members , […]  The complex reveals two helix-turn-helix ( HTH ) motifs within Rob 's N-terminal domain , an arrangement similar to the MarA protein 13 , […]  The Rob protein consists of an N-terminal-DNA-binding-domain that is homologous to the MarA protein 13 , […]  Unlike MarA , Rob does not utilize its C-terminal HTH motif to interact sequence specifically with the major groove of the B-box in the crystal structure […]  The tandem HTH motifs of Rob and MarA exhibit a root mean square ( r.m.s. ) deviation of 0.9 Å , […]  However , there is a striking difference in the interaction of the C-terminal HTH motif with DNA in the Rob and MarA complexes […]  Residues in the N-terminal HTH motif of Rob and MarA that contact conserved bases in the major groove of the A-box are highlighted in blue .  A highly conserved cluster of polar residues in the C-terminal HTH motif ( shaded red ) contact the bases and phosphodiester backbone of the B-box in the MarA -- DNA complex 13 .  […] located on helix αF of MarA 's C-terminal HTH motif , forms direct hydrogen bonds with two guanines ( corresponding to Gua 15 and Ade 7 ´ of micF ; Fig. 1a ) in the major groove adjacent to the B-box sequence .  The remaining contacts between MarA 's C-terminal HTH motif and the B-box of the mar promoter […]  This bulky substitution is located in the major groove of the B-box and it would hinder the C-terminal HTH  [...]  From X-ray crystallographic analysis of the cocrystal of MarA with the marbox from the marRAB promoter , two helix-turn-helix ( HTH ) motifs were identified that make 34 contacts with the DNA and bend it by 35 ° ( 36 ) .  An analysis of MarA by the method of Chou and Fasman ( 4 ) identified two potential helix-turn-helix DNA-binding domains  […]  MarA , SoxS and Rob , activate a common set of promoters which results in multiple antibiotic resistance , superoxide resistance and organic solvent tolerance ( for review , see Alekshun and Levy , 1997 ) .  […]  The marA site involved in the expression of chromosomal multiple antibiotic resistance ,  […]  ; tolerance to antibiotics , organic solvents , and heavy metals ( AarP from Providencia stuartii , MarA and Rob from E. coli , PqrA from Proteus vulgaris , and RamA from Klebsiella pneumoniae ) […] |
| […] Eliminated fragments to save space. |

| **Table 24. Comparison of the manual and automatic summary of ArgR** |
| --- |
| **Manual summary** |
| ArgR has two domains : the N-terminal domain , which contains a winged-helix-turn-helix DNA-binding motif and the C-terminal domain , which contains a motif that binds L-arginine and a motif for oligomerization . Based on cross-linking analysis of wild-type and mutant ArgR proteins , it has been shown that the C-terminus is more important in cer/Xer site-specific recombination than in DNA-binding .  ArgR complexed with L-arginine represses the transcription of several genes involved in biosynthesis and transport of arginine , transport of histidine , and its own synthesis and activates genes for arginine-catabolism . ArgR is also essential for a site-specific recombination reaction that resolves plasmid ColE1 multimers to monomers and is necessary for plasmid stability . |
| **Automatic summary** |
| Results The domain structure of ArgR The mutagenesis results of two laboratories have shown that the ArgR subunit is made up of two functional regions : a basic N-terminal half responsible for DNA-binding and an acidic C-terminal half responsible for both oligomerization and for binding arginine ( Burke et al. , 1994 ; Tian & Maas , 1994 ) .  We overexpressed the C-terminal domain of ArgR ( ArgRc ) corresponding to amino acids 80 to 156 in a T 7 polymerase-driven system and purified the protein to homogeneity .  Discussion The C-terminal domain of ArgR forms a hexameric protein core that contains the binding sites for L-arginine and provides a central , symmetric scaffold for six DNA-binding domains .  In addition to regulating the transcription of arginine biosynthetic genes , ArgR plays an obligatory role in a site-specific recombination reaction that resolves ColE1-like plasmid multimers to monomers and is necessary for plasmid stability ( Stirling et al. , 1988 ) . |

## Figures


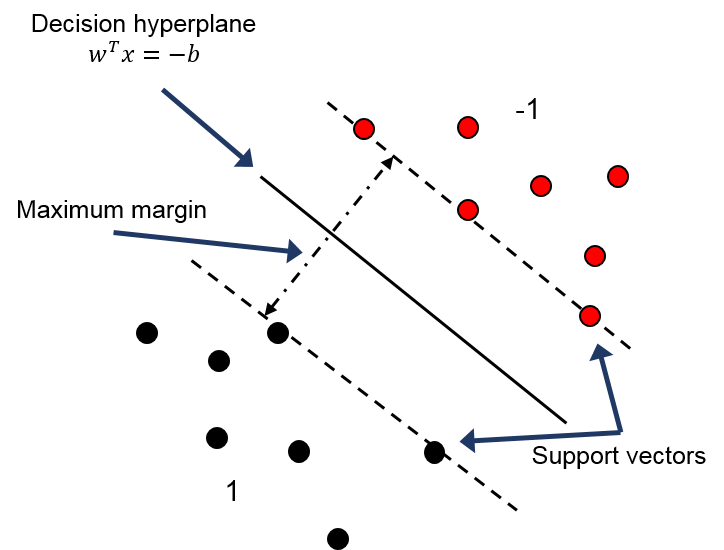


Figure 10. Example of an SVM decision hyperplane.


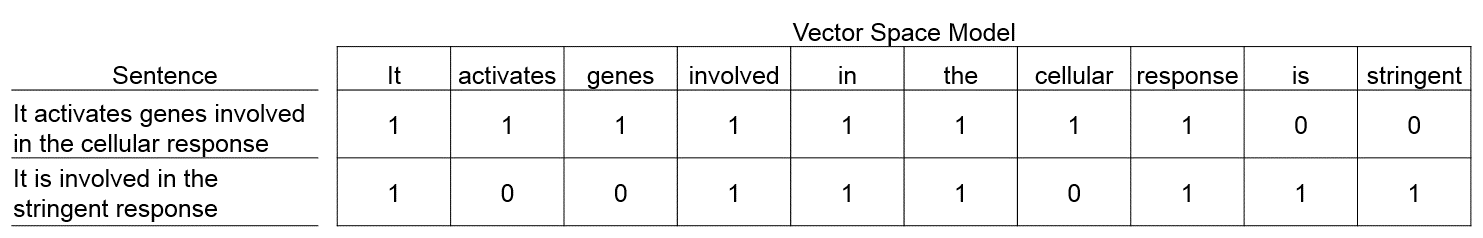


Figure 11. Example of a Vector Space Model.
